# EEG-based deep learning estimates preterm infants’ cerebral age: Contribution of slow-wave and cross-frequency coupling

**DOI:** 10.1016/j.isci.2026.118052

**Published:** 2026-10-26

**Authors:** Alban Gallard, Alexander Billiot, Émilie Bourel-Ponchel, Pauline Brunel, Christèle Chazal, Guy Kongolo, Ghida Ghostine, Fabrice Wallois, Laura Routier, Sahar Moghimi

**Affiliations:** 1 GRAMFC, Inserm UMR1105, Université de Picardie Jules Verne, Amiens, France; 2 Explorations fonctionnelles du Système nerveux Pédiatrique, Centre Hospitalier Universitaire Amiens-Picardie, Amiens, France

**Keywords:** age prediction, convolutional neural network, phase-amplitude coupling, preterm newborn

## Abstract

Reliably estimating cerebral maturation in preterm newborns is crucial for early identification of atypical neurodevelopment. We developed a deep-learning framework using a convolutional neural network (CNN) to predict postmenstrual age (PMA) from spontaneous neural activity in EEG recordings of 165 healthy preterm infants (28–38 weeks PMA). The model achieved a mean absolute error of 0.75 ± 0.60 weeks, with 73.9% of prediction errors within one week. To identify key neurophysiological drivers, we systematically manipulated signal components. Results showed that removing low-frequency activity (<4 Hz) significantly degraded performance and disrupting phase-amplitude coupling selectively impaired prediction accuracy before 34 weeks PMA, while high-frequency activity (>12 Hz) had minimal impact. Our results demonstrate that maturation signatures are primarily driven by slow-wave dynamics and their hierarchical coupling with faster oscillations, which are fundamental hallmarks of early neurodevelopment.

## Highlights

• A CNN model estimated postmenstrual age from bursts of 165 preterm newborns
• Removing low-frequency (<4 Hz) severely degraded prediction
• Removing high-frequency (>12 Hz) had minimal effect on prediction accuracy
• Disrupting phase-amplitude coupling impaired prediction before 34 weeks postmenstrual age

## Graphical abstract

**Figure:**
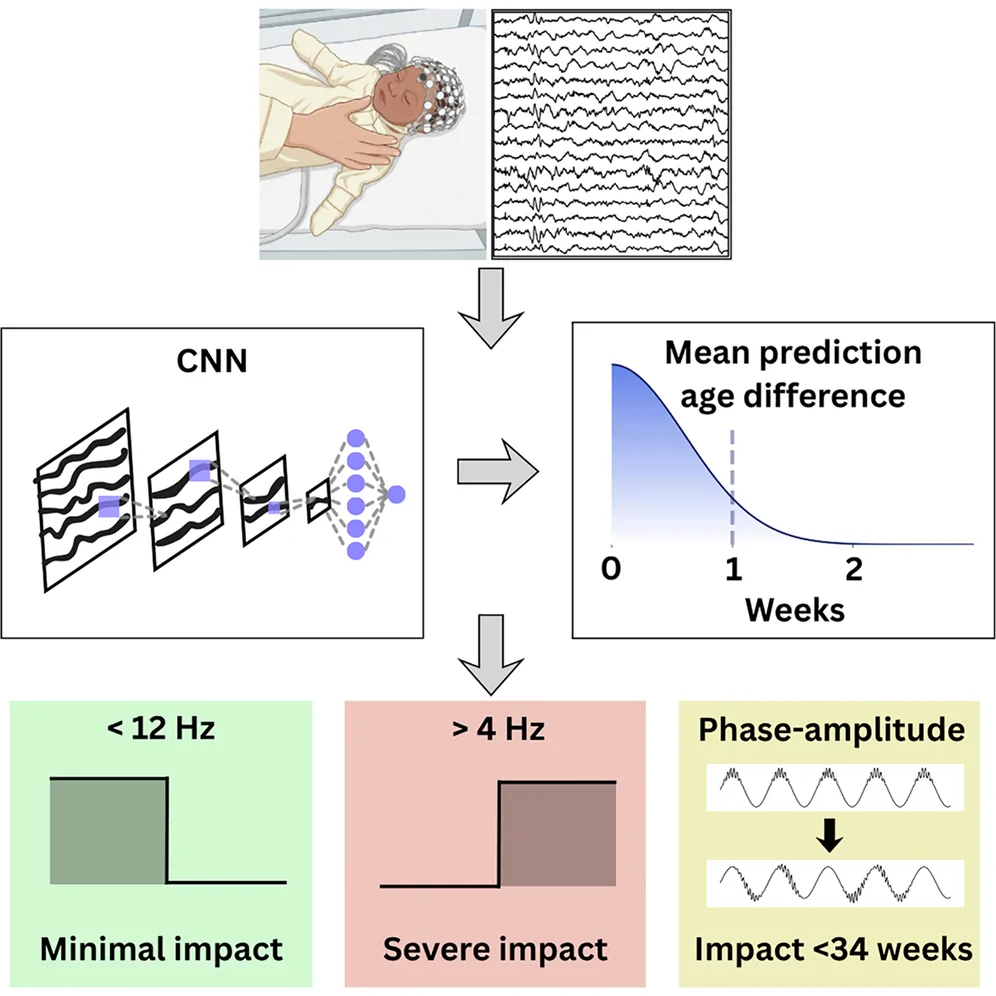

## Teaser

Systems neuroscience; Cognitive neuroscience

## Introduction

During the past 40 years, examining cortical activity in preterm newborns in relation to their gestational age has provided valuable neurophysiological insights into very early neurodevelopment.^1^^,^^2^^,^^3^^,^^4^^,^^5^^,^^6^^,^^7^ Reliable evaluation of cerebral maturation and early detection of deviations from the expected maturational trajectory go beyond theoretical and fundamental research. They have critical practical implications, providing an opportunity for timely and personalized interventions during a period of heightened neuroplasticity to reduce neural/cognitive deficits and disabilities. This requires robust, explicable models capable of capturing the dynamics of cerebral maturation during this period.

During the third trimester of gestation, the human brain undergoes rapid and profound structural and functional transformations.^3^^,^^8^^,^^9^ Neural activity gradually evolves from sporadic, discontinuous bursts toward more organized and sustained patterns reflecting the progressive establishment and maturation of thalamocortical and cortico-cortical networks.^10^^,^^11^^,^^12^^,^^13^ The development of cortical and subcortical functional interactions leads to the emergence and modulation of different oscillatory regimes. These in turn impact the content and characteristics of electroencephalography (EEG) signals.^14^

EEG provides a non-invasive, real-time window into neural activity and constitutes the clinical reference for estimating the progression of cortical maturation. As the brain develops, the EEG background shows hallmark maturational changes including increased continuity, emergence of age-specific patterns (e.g., theta activity with a slow wave, delta brushes, etc.),^1^^,^^2^^,^^14^ and differentiation of vigilance states.^15^ The temporal, spatial and spectral properties of these dynamic EEG patterns provide valuable insights into functional cerebral maturation^16^^,^^17^^,^^18^ and give prognostic information regarding the long-term neurodevelopmental outcomes of preterm infants.^19^^,^^20^

Despite being the current clinical standard for qualitative assessment of cerebral maturation and helping in the estimation of age, visual EEG interpretation has limited precision and reproducibility. Even among expert neurophysiologists, the estimation of EEG-derived cerebral age carries a substantial mean prediction error of ±12 days.^21^ This value reflects population-level prediction performance and inter-individual variability, rather than a deterministic relationship between EEG maturation and chronological age. This limited accuracy arises from the inherently complex, multidimensional, and age-dependent nature of EEG signals, which contributes to substantial inter-rater variability and underscores the need for objective and quantitative alternatives.^21^^,^^22^ Automated analytical approaches—particularly artificial intelligence-based methods—have gained considerable attention for their ability to capture complex developmental signatures and extract highly discriminative neurophysiological features from EEG. Such tools have demonstrated their potential for assessing cerebral function and its maturation from infancy to adulthood^23^^,^^24^ as well as in preterm and full-term newborns.^21^^,^^25^^,^^26^^,^^27^^,^^28^ Despite these advances, it remains necessary to better understand the underlying mechanisms and EEG features driving model decisions. Developing interpretable models is essential not only to improve trust and facilitate clinical implementation, but also to identify the EEG features that underpin cerebral maturation, thereby advancing our understanding of early brain development.

We propose an EEG-based cerebral maturation chart that captures the characteristics of bursts of spontaneous neural activity across the third trimester of gestation equivalent, between 28 and 38 weeks of postmenstrual age (wPMA). To this end, we developed a convolutional neural network (CNN) architecture that encodes burst period of spontaneous neural activity to provide estimates of PMA. Furthermore, to understand the decision of the model, we implemented a data-manipulation strategy to extract the key EEG features underlying maturational stage discrimination.

## Results

Among 165 preterm newborns included in our study, 53% (*n* = 87) were females. EEG recordings were performed with a mean interval of 1.9 ± 1.7 weeks between birth (31.3 ± 1.9 weeks of gestation) and recording (33.1 ± 2.0 wPMA), yielding the PMA distribution shown in Figure 1A. Gestational age at birth was estimated from a first-trimester dating ultrasound. Mean birth weight was 1,480 ± 390 g and mean weight at the time of EEG recording was 1,600 ± 390 g. More information about the participant demographics is presented in Table S1 of supplementary information.

**Figure 1:**
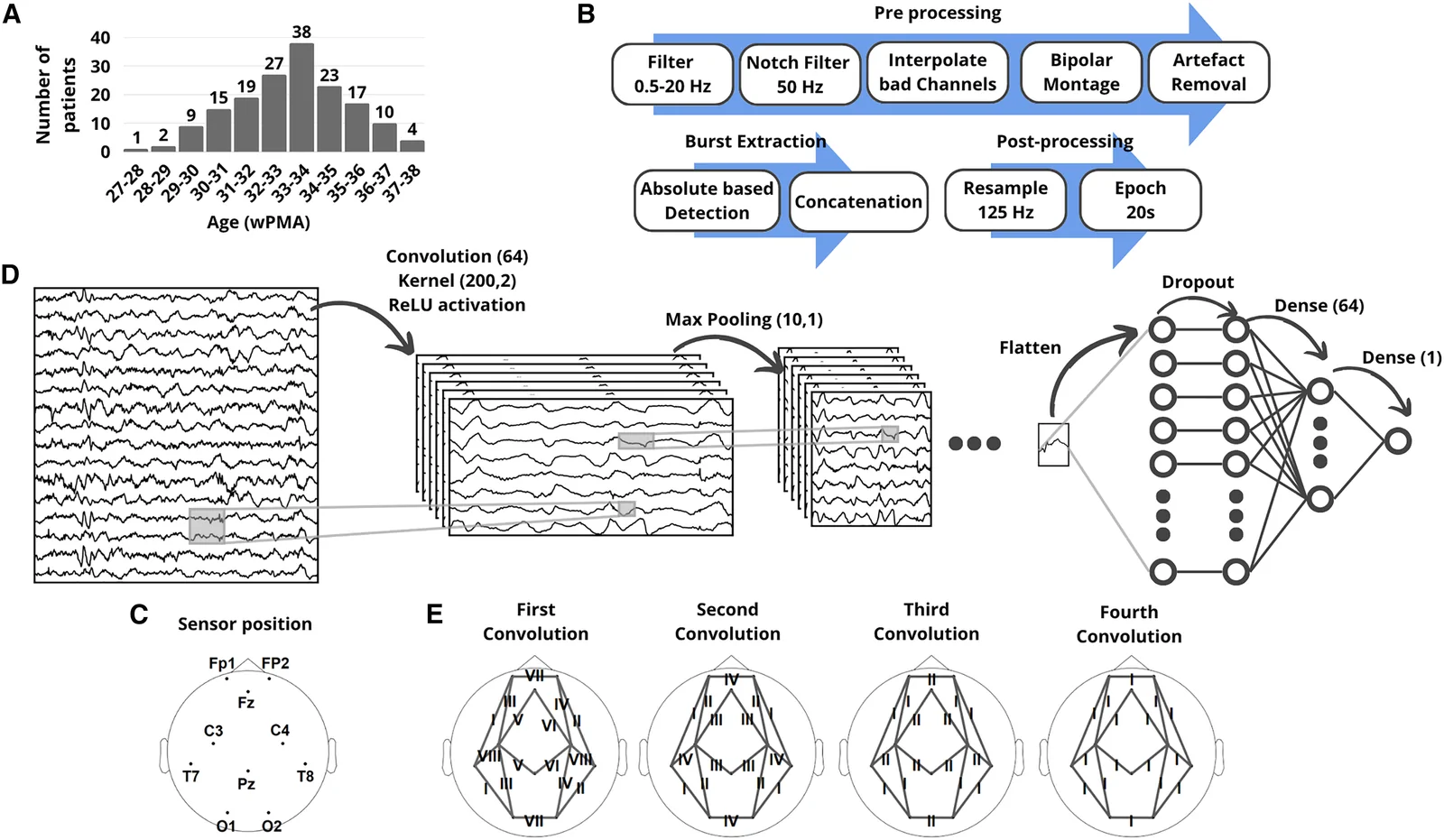
Study design (A) Distribution of the participants: the distribution of the 165 newborns included across postmenstrual age (28–38 wPMA) at the time of recording. (B) EEG preprocessing pipeline: steps for preparing the acquired raw signals into 20-s epochs for the CNN. The procedure is divided into three categories: preprocessing, burst extraction, and post-processing. (C) CNN architecture: the model was trained using 10-fold cross-validation to predict the PMA of the preterm newborns represented in (A). The 20-s EEG burst windows were fed into the first layer, passing through four blocks-each comprising one convolution layer and one pooling layer. For clarity, only the first block is detailed in this diagram, featuring a convolution layer of 64 kernels (size 200 × 2), a ReLU activation, and a max-pooling layer with a 10 × 1 window. The blocks are separated with a normalization layer. The network concludes with a flatten layer, a dropout layer and two dense layers. (D) Sensor configuration: position of the sensors used to create the custom bipolar montage. (E) Spatial representation of convolutions: illustration of the convolutions across the four blocks; for instance, the two bipolar channels on the left, numbered I (equivalent of Fp1-T7 and T7-O1) are convolved with the 64 kernels of the first convolution layer.

After preprocessing the EEG data, bursts of spontaneous activity were detected (Figure 1B). The mean burst duration across newborns was 7.82 ± 1.61 s, giving a total of 17,221 epochs of concatenated 20-s bursts of activity extracted from 165 preterm EEG (104 ± 68 epochs per newborn). All EEG data were transformed to a custom 16-channel bipolar montage (Figure 1C). The CNN model (Figures 1D and 1E) was trained with a 10-fold cross-validation. The training and testing folds were composed of 15,499 ± 230 epochs (range: 15,063–15,759) and 1,722 ± 230 epochs (range: 1,462–2,158), respectively. The model was then evaluated based on uniform manifold approximation and projection (UMAP) and prediction performance. Finally, EEG data were manipulated in three different scenarios targeting: *(i)* the high-frequency content (>12 Hz); *(ii)* the low-frequency content (<4 Hz); and *(iii)* the interaction between the low- and high-frequency contents in the bursts of neural activity. The impact of manipulations and prediction accuracies were evaluated at different PMAs and in different subgroups: 28–32 wPMA, 32–34 wPMA, and 34–38 wPMA.

### UMAPs analysis

The UMAPs presented in Figure 2A show the evolution of network activation across the blocks—i.e., a convolutional layer followed by a pooling layer—progressing from a random pattern in the first block toward a systematic organization according to PMA in the final block. This progression ultimately resulted in distinct clustering of bursts as a function of PMA.

**Figure 2:**
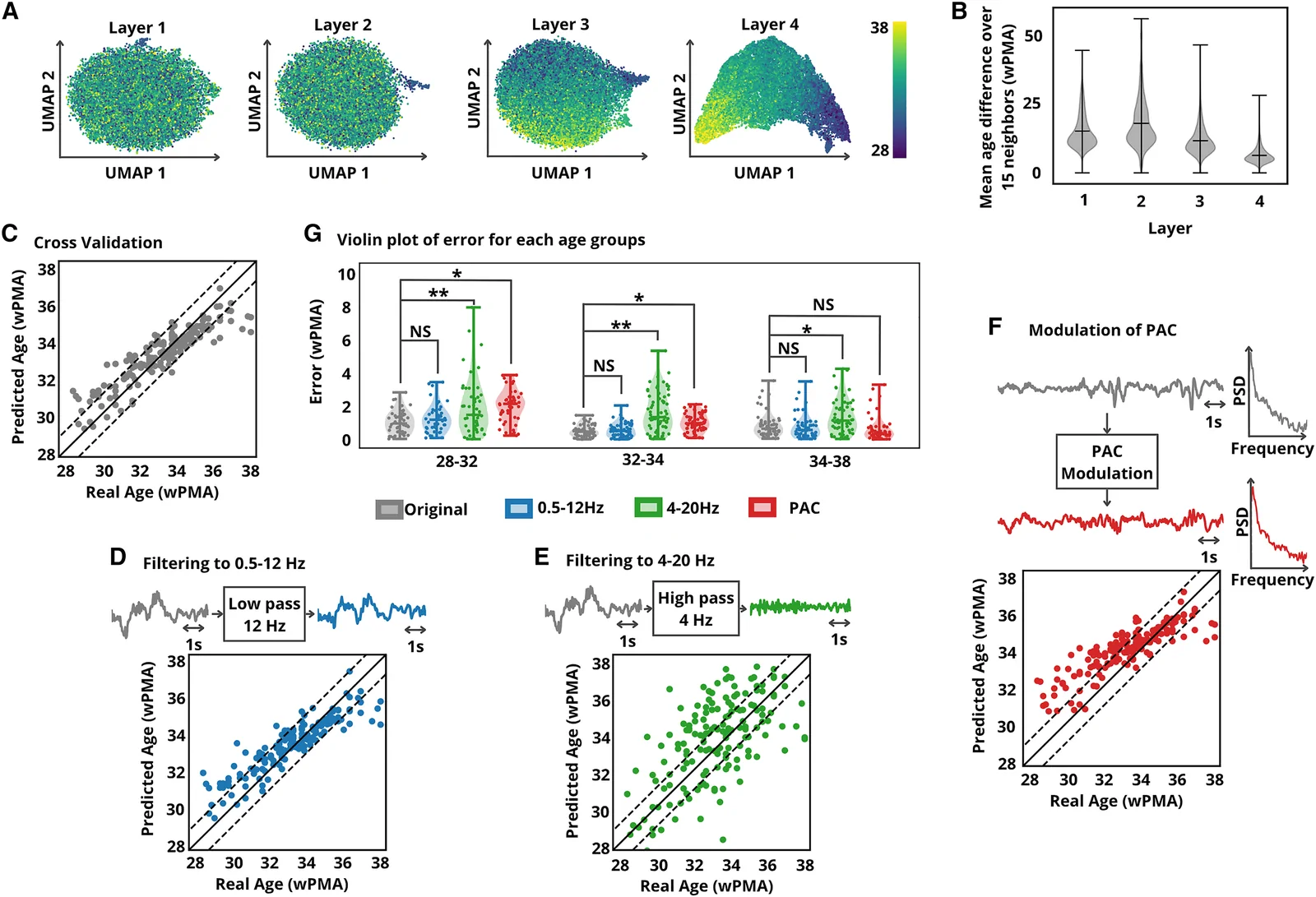
Prediction of cerebral maturation and impact of EEG characteristics (A) Lower-dimensional representation of learning within CNN layers: each dot represents a 20-s epoch, with all participants pooled together; color coding indicates the postmenstrual age (PMA) at the time of recording. (B) Layer-wise impact analysis: the impact of each layer was investigated by calculating the mean age difference over 15 neighbors. Violin plots display the mean age difference for each point after each layer. (C) Cross-validation results: 10-fold cross-validation results for EEG-based prediction of cerebral age. The x axis represents the PMA at the time of recording, validated by a clinician. The y axis represents the predicted age by the network. Each dot represents a newborn, with a predicted age difference of 0.75 ± 0.60 wPMA. The solid line represents the line of identity (R^2^ = 0.77). The dashed line represents the ± one-week error boundary, with 73.9% of predictions falling within this range. (D–F) Impact of signal manipulation: relationship between predicted and real age for each newborn after: (D) removing high-frequency content (filtered at 0.5–12 Hz), resulting in a mean absolute error (MAE) of 0.78 ± 0.67 wPMA, R^2^ = 0.74 and 73.3% predictions within one week; (E) removing low-frequency content (filtered at 4–20 Hz), with MAE = 1.58 ± 1.26 wPMA, R^2^ = 0.008 and 38.2% predictions within one week; and (F) disrupting the phase-amplitude relationship between low- and high-frequency activity, resulting in MAE = 1.09 ± 0.87 wPMA, R^2^ = 0.53 and 56.4% predictions within one week. (G) Subgroup analysis of modulation: The impact of each modulation was further investigated in three age groups: 28–32 wPMA, 32–34 wPMA, and 34–38 wPMA. Violin plots show the prediction error for each age group on the cross-validation data as well as the three modulation scenarios (D–F). Each dot represents the prediction error for a newborn. NS: non-significant difference (*p* > 0.05), ∗: *p* < 0.05, and ∗∗: *p* < 0.005. In all graphs, gray represents the original data, blue indicates data filtered between 0.5–12 Hz, green indicates data filtered between 4–20 Hz, and red represents the disruption of phase-amplitude coupling.

For further analysis, the Pearson correlation between the distance of each data pair (148,272,810 pairs) and their absolute difference in PMA was calculated for each block. The correlation increased after each block: r = 0.005 (*p* < 0.0001) for the first block, r = 0.018 (*p* < 0.0001) for the second block, r = 0.21 (*p* < 0.0001) for the third block, and r = 0.70 (*p* < 0.0001) for the fourth block.

The mean age difference among neighbors began to decrease after the second block (Figure 2B): 15.3 ± 6.4 wPMA for the first block, 18.1 ± 9.1 wPMA for the second block, 11.7 ± 5.2 wPMA for the third block, and 6.4 ± 3.0 wPMA for the fourth block. The ANOVA on the mean age difference among neighbors revealed a significant main effect of block (F[3, 68,880] = 10,889, *p* < 0.0001, ƞ_p_^2^ = 0.32). Tukey’s Honest Significant Difference post-hoc analysis, detailed in Table S2 of supplementary information, indicated significant difference between all pairs of blocks. This analysis indicated that grouping of epochs according to PMA differed significantly between blocks.

### Prediction of postmenstrual age

After cross-validation results, 54.6% and 85.2% of epochs were predicted with an age difference of less than one week and less than two weeks, respectively. The mean predicted age difference was 1.10 ± 0.90 wPMA. Averaging over epochs for each newborn improved the prediction accuracy; PMA was predicted within a one-week range for 73.9% of newborns and within a two-week range for 95.2% of newborns. The mean predicted age difference at the population level was 0.75 ± 0.60 wPMA (R^2^ = 0.77). Cross-validation results at population level are shown in Figure 2C. The mean predicted age difference at the population level (0.75 ± 0.60 wPMA) was smaller than a predefined baseline, corresponding to the mean difference between each participant’s age and the mean population age (33.1 wPMA), which was equal to 1.61 ± 1.23 wPMA.

After dividing the population into three subgroups, the mean predicted age difference was 1.01 ± 0.62 for 28–32 wPMA, 0.51 ± 0.35 for 32–34 wPMA, and 0.81 ± 0.71 for 34–38 wPMA. Additionally, the prediction of PMA was within a one-week range for 51.1% of newborns in the 28–32 wPMA subgroup, 90.6% in the 32–34 wPMA subgroup, and 74.1% in the 34–38 wPMA subgroup. This indicates superior prediction accuracy for newborns between 32 and 34 wPMA compared to the other subgroups. The ANOVA on the prediction error revealed a significant main effect of age group (F[2, 162] = 10.64, *p* < 0.0001, ƞ_p_^2^ = 0.12). Post-hoc analysis, detailed in Table S2 of supplementary information, indicated significant differences between the middle group and the two other groups, but not between the youngest and oldest groups.

### EEG manipulations

Modulation of the high frequency content, *scenario (i)*, slightly increased the mean prediction difference, as shown in Figure 2D, with a 0.78 ± 0.67 predicted age difference (R^2^ = 0.74) and 73% of preterm newborns predicted with a difference of less than one week. Filtering the data between 4 and 20 Hz, *scenario (ii)*, resulted in a more drastic increase in the predicted age difference (1.58 ± 1.26, R^2^ = 0.008) and a significant reduction of the percentage of newborns with a prediction difference of less than one week (38%), as shown in Figure 2E. In *scenario (iii)*, disrupting phase amplitude coupling (PAC) increased the predicted age difference (1.09 ± 0.87, R^2^ = 0.53) and decreased the number of newborns predicted with an error of less than one week to 56% (Figure 2F).

The ANOVA on the predicted age difference, with scenario as a within-subject factor and age as a between-subject factor, revealed a significant main effect of scenario (F[3, 486] = 49.4, *p* < 0.0001, μ = 24.57, ƞ_p_^2^ = 0.23) and a significant main effect of age (F[2, 162] = 19.3, *p* < 0.0001, μ = 24.97, ƞ_p_^2^ = 0.19). The interaction between the two factors was also significant (F[6, 486] = 6.97, *p* < 0.0001, μ = 3.47, ƞ_p_^2^ = 0.08). Post-hoc analysis (Figure 2G), detailed in Table S2 of supplementary information, further evaluated the impact of the manipulations (scenarios) within each subgroup. For the youngest subgroup (28–32 wPMA), post-hoc analysis indicated no significant impact of *scenario (i)* with a weak BF = 0.446. However, it showed a significant impact for the other two scenarios. For the age group 32–34 wPMA, the impact of *scenario (i)* was not significant, with a moderate BF = 0.195. However, the impact of *scenario (ii)* and that of *scenario (iii)* were significant. For the oldest subgroup (34–38 wPMA), the impact of *scenario (ii)* was significant, whereas those of the other two scenarios were not significant, with a moderate BF = 0.267 for *scenario (i)* and a weak BF = 0.851 for *scenario (iii)*. In summary, these analyses showed that removing the high-frequency content in *scenario (i)* did not significantly impact the prediction accuracy (although a weak BF should be treated with caution). However, removing the low frequency content in *scenario (ii)* significantly impacted prediction accuracy across all age groups. Finally, disruption of PAC in *scenario (iii*) only had a significant impact on the prediction accuracy for age groups below 34 wPMA.

## Discussion

Our CNN achieved strong performance, with 73.9% of infants having a predicted age difference of less than one week, 95.2% having a prediction of less than two weeks, and a mean predicted age difference of 0.75 ± 0.60 weeks. The study further revealed that the proposed model, and potentially other models trained directly on spontaneous neural activity (and not extracted features) at this stage of neurodevelopment, capture the modulation of slow oscillations and their orchestration with faster spontaneous activity, rather than relying on the amplitude of the full spectral content or faster neural activity. In contrast to previous works incorporating the entire EEG signal,^25^^,^^26^ our model deliberately excluded discontinuous periods and instead targeted the bursts of spontaneous activity linked to underlying mechanisms leading to evolving bouts of neural activities. These findings illustrate the capacity of deep learning architectures to extract age-related neurophysiological signatures by encoding both spatial distribution and temporal dynamics of the spontaneous activity in EEG signals.

EEG has proven to be a fundamental and reliable tool for assessing cerebral maturation and neurological risk in preterm newborns, as its temporal, spatial, and spectral characteristics evolve rapidly with increasing PMA and cerebral maturation.^2^^,^^3^^,^^4^ Experimental data show that intermittent bursting is a ubiquitous characteristic of early neural activity and contributes to the development and refinement of neural circuits.^11^^,^^29^^,^^30^ Building on this empirical foundation, the present study demonstrates the strong potential of EEG-based machine learning models—particularly CNNs—for predicting functional cerebral maturation in preterm infants based on the “*content*” of spontaneous activity bursts. Our model accurately estimated EEG cerebral age of preterm infants, achieving performance comparable, in terms of accuracy and prediction under one week, compared to previously published machine-learning approaches during this developmental window.^21^^,^^22^^,^^25^^,^^28^ More precisely, the model closely tracked the expected maturation with a mean absolute error close to one week. This level of precision approached the intrinsic uncertainty in the clinical estimation of PMA.^31^^,^^32^ These results confirm that EEG-based models can reliably capture the maturation of cortical and subcortical functions in early life, despite substantial inter-individual variability.

The signal manipulation experiments provided more insight into the automatic encoding strategy: the model relies predominantly on low-frequency dynamics and their hierarchical coupling with faster oscillations, rather than wide spectral or high-frequency power alone. Removing low-frequency components (<4 Hz) significantly degraded model performance, a coherent result regarding the importance of delta band at this age,^1^^,^^2^^,^^3^ and this effect persisted when the population was stratified into three subgroups representing the early, mid, and late third-trimester periods. In contrast, removing the high-frequency content (>12 Hz) did not significantly affect the model’s performance, either at the population level or within subgroups.

Perturbing the coupling between the phase of slow oscillations and the amplitude of faster oscillations, while preserving power in both bands, led to a significant decrease in prediction accuracy. Interestingly, disrupting PAC had the most pronounced effect for infants between 28 and 32 wPMA, a developmental stage marked by the emergence of nested oscillatory patterns, in which faster oscillations occur at a specific phase of slow waves.^3^^,^^14^ At this stage, these electrical patterns constitute the principal electrophysiological signature of activity bursts. This includes theta oscillations nested in slow waves, first appearing from 24 to 25 wPMA,^33^^,^^34^ and sigma band oscillations nested in slow waves, referred to as delta brush in human EEG, first appearing around 27–28 wPMA,^35^^,^^36^ and spindle burst in rat models.^37^^,^^38^^,^^39^^,^^40^ Beyond these classical neurobiomarkers, slow oscillations modulate the amplitude of faster activity during spontaneous bursts.^41^^,^^42^ These cross-frequency interactions evolve systematically with cerebral maturation.^18^^,^^43^^,^^44^ Such modulation likely reflects both the development of thalamocortical pathways and the maturation of local corticocortical and subplate-cortical circuits. Computational studies suggest that changes in the thalamocortical and hippocampocortical synaptic strength can shape both the morphology of slow oscillations and their phase-amplitude relationships.^45^^,^^46^ While the present study cannot provide direct evidence regarding the underlying networks, our results indicate that the model captures the developmental EEG signatures generated by the underlying neural circuits that lead to more pronounced variation in the characteristics of slow oscillations and phase-amplitude relations at this stage. Notably, the model appears to rely less on phase-amplitude relationships near-term, when neuro-biomarkers characterized by nested oscillations progressively disappear from the neonatal EEG.^1^^,^^2^^,^^3^^,^^4^

Our work demonstrates that accurate estimation of cerebral age is achievable based on low-density EEG and raw activity bursts with the advantage of automatic data preparation. In our proposed CNN-based model, PMA prediction primarily relied on the maturation of low frequency activity and its coupling with faster oscillations, key hallmarks of early functional cerebral development. By focusing exclusively on typically developing preterm infants, this work establishes a normative reference for early functional cerebral maturation. Future work should characterize deviations from this normative trajectory to identify preterm infants at increased risk for adverse neurodevelopmental outcomes. Longitudinal, individual-level EEG monitoring will be essential to delineate developmental trajectories and determine how early pathological events disrupt neurodevelopment and shape long-term outcomes. In this context, the brain-age gap emerges as a promising quantitative marker of early neurodevelopmental divergence. Ultimately, these findings provide a foundation for the development of clinically interpretable artificial intelligence-based tools that combine automated PMA estimation with visualization of the EEG features driving each prediction. Such tools could complement expert visual interpretation by providing an objective measure of cerebral maturation, facilitating longitudinal monitoring of developmental trajectories, and helping clinicians distinguish delayed or atypical maturation from normal developmental variability.

### Limitations of the study

Our study has several limitations. First, sleep-wake states were not considered. Bursts of activity were detected across the entire spontaneous brain activity recording. Although it is unlikely that this affected the developmental trend, a sleep state-specific analysis of bursts (e.g., similar to Iyer^23^ and Moghadam^27^) could provide a more detailed understanding of the evolution of spontaneous neural activity. Moreover, quiet sleep is characterized by less rich and more “immature” EEG patterns than active sleep and wakefulness. Incorporating vigilance-state information could, therefore, reduce inter-participant variability across epochs and potentially improve model sensitivity and accuracy. In the same vein, future studies should also account for the use of sedative and analgesic medications, which may significantly influence neonatal EEG activity and thus contribute to inter-participant variability.^47^ In addition, including participants with more variability in other maternal and neonatal demographic characteristics (like sex or delivery) would be helpful to study the impact of such features on the prediction of PMA.

Secondly, due to clinical and logistical constraints, PMA was not uniformly distributed, resulting in higher predictive performance around the middle of the third trimester of gestation and reduced accuracy at both developmental extremes. A more balanced age distribution will be necessary to mitigate sampling bias and stabilize performance across the full spectrum of prematurity.

Thirdly, although relatively large for neonatal research, our cohort remains modest with respect to current methodological recommendations, which typically advocate for several hundred participants.^48^

These limitations are not ancillary; they define the key steps required before clinical translation. Prior the deployment as a clinically usable tool for the objective and quantitative assessment of functional brain maturation and neurodevelopmental trajectories, such an EEG-based model requires validation in a large, multi-center cohort of preterm newborns to ensure generalization across recording systems, clinical environments and demographic variability. A recent study highlighted the feasibility of longitudinal EEG-based assessment of functional brain maturation in preterm newborns using serial reduced-spatial and temporal resolution EEG recordings combined with deep learning-based cerebral age modeling.^26^ Integrating longitudinal functional brain data derived from repeated EEG recordings acquired throughout the period of prematurity—as recommended in clinical practice^49^^,^^50^—could enable the development of a robust longitudinal framework to accurately characterize neurodevelopmental trajectories in high-risk infants. Such a longitudinal functional approach could ultimately provide a clinically relevant tool for identifying atypical maturational trajectories and infants at increased risk of adverse neurodevelopmental outcomes. This functional longitudinal approach could also be incorporated into a multimodal model including MRI-based structural morphometry, which can be routinely performed at term-equivalent age, and relevant biological markers. Brain MRI has already demonstrated high accuracy for cerebral age prediction.^51^^,^^52^ Such an integrated multimodal strategy can substantially improve our understanding of the physiological mechanisms driving early cerebral maturation as well as the pathological processes that may disrupt it, while enhancing the early detection of atypical neurodevelopmental trajectories.

## Resource availability

### Lead contact

Requests for further information and resources should be directed to and will be fulfilled by the lead contact, Alban Gallard (alban.gallard@u-picardie.fr).

### Materials availability

This study did not generate new unique reagents.

### Data and code availability

• The data reported in this study cannot be deposited in a public repository because of participants not providing consent to share their data outside our research consortium on the consent form. To request access, contact Alban Gallard (alban.gallard@u-picardie.fr).
• All original code has been deposited on this GitHub repository: https://github.com/AlbanGallardGramfc/EEG-based-functional-cerebral-maturation-modelduring-the-third-trimester-of-gestation.
• Any additional information required to reanalyze the data reported in this study is available from the lead contact upon request.

## Acknowledgments

We thank Amiens University Hospital EEG technicians for data acquisition and the parents and families who consented to take part in the study. This work was supported by funding from the 10.13039/501100001665French National Research Agency under Grants ANR-22-CE45-0034 (HINT) and ANR226CPJ1-0067-01.

## Author contributions

A.G.: conceptualization, formal analysis, methodology, software, validation, visualization, and writing – original draft; A.B.: conceptualization, investigation, methodology, software, and validation; É.B.-P.: data curation, resources, writing – review and editing; P.B.: investigation and resources; C.C.: resources and writing – review and editing; G.K.: resources and writing – review and editing; G.G.: resources and writing – review and editing; F.W.: funding acquisition, resources, and writing – review and editing; L.R.: conceptualization, data curation, resources, and writing – original draft; S.M.: conceptualization, funding acquisition, project administration, supervision, validation, and writing – original draft.

## Declaration of interests

The authors declare no competing financial interests.

## STAR★Methods

### Key resources table

**Table:** 

REAGENT or RESOURCE	SOURCE	IDENTIFIER
**Deposited data**		

Code	This paper	https://github.com/AlbanGallardGramfc/EEG-based-functional-cerebral-maturation-modelduring-the-third-trimester-of-gestation

**Software and algorithms**		

Python 3.12.6	Python Software Foundation	https://www.python.org

### Experimental model and study participant details

A total of 165 preterm newborns participated in our study with a PMA range of 28–38 weeks at recording, with 87 female (53%). The distribution of PMA at the time of EEG recording is shown in Figure 1A. More information about the participant demographics is presented in Table S1 of supplementary information. In total, 73.5 hours of EEG (with a mean of 24.8 ± 16.9 min per newborn) were recorded during sleep in the neonatal intensive care unit of the Amiens-Picardie University Hospital, France. All participants met the following inclusion criteria: preterm birth between 24 and 37 weeks of gestation, age at recording between 28 and 38 wPMA, birth weight, size, and head circumference appropriate for PMA at the time of EEG recordings, normal clinical and neurological assessments at the time of EEG recording, and no severe intracranial lesions (high-grade intraventricular haemorrhage or periventricular leukomalacia) on cranial ultrasound. Exclusion criteria included scalp dermatological lesions, venous access devices placed on the scalp, or any scalp injury preventing EEG electrode placement. Follow-up EEG evaluations were performed and were required to be normal, in accordance with the guidelines of the French Society of Clinical Neurophysiology.^49^^,^^50^ Both parents were informed about the study and provided written informed consent. The study was approved by the ethics committee (Comité de Protection des Personnes Ouest, ID-RCB: 2019-A01534-53).

### Method details

#### EEG recording and processing

EEG signals were recorded using either a 128-channel or a 64-channel HydroCel Geodesic Sensor Net (depending on the head circumference), with an Electrical Geodesic NetAmps 200 amplifier passing a digitised signal to Electrical Geodesics Net Station software (version 5). EEG was digitised at a 1,000 Hz sampling rate, using a Cz vertex electrode as reference.

The recorded signals were processed in Python (MNE and custom functions) following the steps outlined in Figure 1B. A zero-phase FIR filter was applied on raw EEG signals with a Hamming window and an order equivalent to 3 cycles of the low-frequency cutoff (0.5 to 20 Hz), resulting in a filter length of 6000 samples at a 1000 Hz sampling rate. Additionally, an Infinite Impulse Response 50 Hz notch filter was applied to remove the very large power line interference. Subsequently, bad channels were identified with the Local Outlier Factor algorithm^53^^,^^54^ and interpolated using the spherical spline method.^55^ Finally, with the goal of developing a clinical tool in future studies, 10 electrodes were kept and EEG signals were re-referenced to a custom 16-channel bipolar montage (Figure 1C): Fp1-T7, T7-O1, Fp1-C3, C3-O1, Fp2-T8, T8-O2, Fp2-C4, C4-O2, Fz-C3, C3-Pz, Fz-C4, C4-Pz, Fp1-Fp2, O1-O2, T7-C3 and C4-T8. EEG segments containing artefacts were identified and excluded using amplitude and first derivative thresholds. More precisely, any time point exceeding an absolute amplitude value of 400 μV was marked as artefact and discarded, together with a 2-second window before and after the detected artefact. Next, the first derivative at each point was calculated as the difference between consecutive samples. One-second segments with an average of the derivative above 400 μV/sample were marked as artefact and removed from further analyses.

Artefact-free EEG data were then processed to detect bursts of neural activity. We limited the analyses to the periods of bursts to better understand the dynamics of spontaneous periods and their link with cerebral maturation. The absolute amplitude of signals was computed for each channel. Next, the 40^th^ and 80^th^ amplitude percentiles were calculated independently per channel. These two thresholds were manually optimised through repeated evaluation. At each channel location, a sample was preselected if its absolute amplitude exceeded the 40^th^ percentile in at least six (out of the 16) bipolar channels. Among these preselected periods, a burst was confirmed when at least one sample exceeded the 80^th^ percentile in at least one channel. Burst periods shorter than 1 s were discarded and those separated by inter-burst intervals shorter than 1 s were merged. All burst periods were zero-meaned and concatenated with a local smoothing at the edge (cosine taper) to avoid spurious effects, resulting in a continuous signal, thereby excluding inter-burst intervals from subsequent analyses. To ensure sufficient data, participants with less than 1 min of burst of spontaneous activity were to be excluded from further analysis. No participant fell below this threshold, so none were excluded on this basis.

During post-processing, the signals were downsampled to 125 Hz and segmented into 20-second epochs with 10-second overlapping, resulting in a total of 17,221 epochs to train the CNN (104 ± 68 epochs per newborn). We segmented the data into 20-second windows to balance dataset size with signal length. While longer epochs yield a smaller training set, shorter epochs risk capturing insufficient information for robust analysis.

#### Convolutional neural network training and testing

The model was based on a CNN, which used kernels that could slide across the time axis to code the temporal dynamics at multiple scales and across channels to capture spatial correlations between different brain regions. In similar scenarios, CNN has a better performance with minimal data compared to more recent methods such as Transformers.^56^^,^^57^^,^^58^ The CNN architecture, detailed in Figure 1D, consisted of four blocks, each containing a convolutional layer with a ReLU activation function followed by a pooling layer.^59^^,^^60^ More precisely, the first block was composed of a convolutional layer with 64 kernels of size 200 × 2 and a ReLU activation followed by a max-pooling layer with a 10-sample pooling window. In the remaining three blocks, the convolutional layer had 32, 16, and 8 kernels respectively, of size 10 x 2 with a ReLU activation. Similar to the first block, the second block included a max-pooling layer with a 10 × 1 window. The third block utilised an average-pooling layer (10 × 1), and the final block had a global average-pooling layer. The convolutional layer of each block encoded the spatial distribution of the neural activity over the montages shown in Figure 1E. A temporal z-scoring was performed between every two blocks.^61^ The output from the final pooling layer was then flattened into a one-dimensional array, which was followed by a dropout layer to prevent overfitting.^62^ Finally, two consecutive fully connected (dense) layers of sizes 64 and 1 (Figure 1D) generated the output,^63^ here predicting the PMA. The architecture of the CNN was decided after a series of training and testing steps that led to the aforementioned optimal structure.

The CNN was trained with a stratified 10-fold cross validation at the population level, ensuring that: (1) no epoch from a left-out participant was seen by the model during training; and (2) fold distribution was relatively similar between each fold by selecting the same ratio of newborns in each age groups. The loss function to be minimised by the CNN during the training process was the mean square difference between the predicted and real PMA.

The prediction was then evaluated by calculating: (1) the predicted age difference (absolute error between the predicted and real PMA) for each epoch; and (2) the percentage of epochs with a predicted age difference of less than one week. To obtain a single maturation estimate for each newborn, the predictions for all epochs belonging to that individual’s EEG recording were averaged. The line of identity was plotted at the population level, and the associated coefficient of determination (R^2^) was calculated. For further evaluation, a baseline was defined as the mean difference between each participant’s age and the mean cohort age (33.1 wPMA). Next, the global model prediction error (mean predicted age difference) was compared with this baseline value.

#### Model analysis

First, to visualise and evaluate learning in the progression of blocks within the CNN, the unseen data corresponding to each fold of the cross validation were used as input to the CNN and the output of each block was calculated. The UMAP of activation was calculated with 15 neighbours for the output of each block to reduce high-dimensional representation at each network activation.^64^ The first two UMAPs components were retained for further evaluation.

Next, for each block, the two-dimensional distance between every pair of data points and the absolute difference of the corresponding PMA were calculated. The Pearson correlation between the distance and the difference in PMA values was calculated. Moreover, to evaluate the organisation of the points after each block, the difference in PMA was calculated between each point and its 15 neighbours and averaged. To investigate the impact of each block on the predicted age difference, we conducted a one-way analysis of variance (ANOVA) on the mean age difference with the neighbours, with the factor block. This was followed by a Tukey's Honest Significant Difference post-hoc analysis, providing evidence on progressive encoding over the blocks toward the EEG-based prediction of cerebral age.

To further evaluate the model’s ability to predict maturation across different gestational periods, we divided the population into three subgroups with similar number of newborns: 28 wPMA - 31 wPMA + 6 d (*N* = 46), 32 wPMA - 33 wPMA + 6 d (*N* = 65), and 34 wPMA - 37 wPMA + 6 d (*N* = 54). To improve readability, these groups are referred to as 28-32 wPMA, 32-34 wPMA, and 34-38 wPMA, respectively. These groups were broadly aligned with standard clinical PMA categories. Mean predicted age differences were calculated at the subgroup level for further statistical analysis. To investigate prediction accuracy over each age group, we conducted a one-way ANOVA on the prediction errors with factor age (28-32 wPMA, 32-34 wPMA, and 34-38 wPMA). This was followed by a Tukey's Honest Significant Difference post-hoc analysis. This provided evidence of the model’s accuracy within each age range.

#### Modulations

Explaining the learning process of a CNN model is a difficult task. To investigate some features learnt by the model to predict PMA, we manipulated the test data in each fold during the cross-validation phase, while keeping the trained CNN intact. These manipulations do not fully explain the learning process but give us insight into EEG features learnt by the model and consequently linked with cerebral age. The manipulations were carried out in three different scenarios targeting: *(i)* the high frequency content; *(ii)* the low frequency content; and *(iii)* the interaction between the low- and high- frequency contents in the bursts of neural activity. In *scenario (i),* the data were band-pass filtered between 0.5 and 12 Hz (filter order: 3 cycles of the low-frequency cut-off) to evaluate the effect of removing the higher-frequency content specific to bursts of activity, especially after 28 wPMA, as well as specific neurodevelopmental biomarkers such as delta brushes.^3^^,^^35^^,^^65^ In *scenario (ii)*, the data were band-pass filtered to 4-20 Hz (filter order, 3 cycles of the low-frequency cut-off) to evaluate the impact of losing the lower-frequency content of bursts. These components correspond to slow delta-band oscillations, a characteristic that shows systematic modulation with neurodevelopment.^1^^,^^66^ Finally, in *scenario (iii)*, we disrupted the phase-amplitude relationship (PAC), a hallmark characteristic of spontaneous neural activity in early and later developmental stages.^18^^,^^33^^,^^67^ If the modification of phase-amplitude relations between slow and fast activates impacts the results, it indicates the importance of this characteristic for the model to predict PMA. To this end, we computed the Fourier transform of each epoch and randomly replaced the phase of frequencies below 4 Hz with that of another epoch, therefore disrupting the phase-amplitude relationship, without modifying the power spectral density. The manipulated time series of each epoch were then created using the inverse Fourier transform. To quantify and compare the impact of each manipulation, the mean predicted age difference, R^2^ relative to the line of identity, and the percentage of prediction differences within one week were calculated and subjected to further statistical analysis.

To investigate the impact of modulations in *scenarios (i)*-*(iii)* on the prediction of cerebral maturation, we conducted a 4 × 3 ANOVA. Scenario was treated as a within-subjects factor [Original, 0.5-12Hz, 4-20Hz, and PAC], while age was a between-subject factor [28-32 wPMA, 32-34 wPMA, and 34-38 wPMA]. This was followed by a Tukey's Honest Significant Difference post-hoc analysis for the factor “scenario”, carried out separately within each age subgroup. This provided evidence regarding the importance of each characteristic as a function of PMA. When necessary, we employed Bayesian t-test statistics to derive a Bayes factor, providing an unbiased decision concerning the null hypothesis.^68^

### Quantification and statistical analysis

All statistical analysis were performed with Python. The first statistical analysis was the comparison of UMAPs explained in subsection “model analysis” and the results shown in subsection “UMAPs analysis”. The statistical analysis on the effect of age on the prediction of PMA is explained in subsection “model analysis” and the results are shown in subsection “prediction of postmenstrual age*”. The statistical analysis on the effect of modulations is explained in the subsection “*modulations*” and the results are shown in* Figure 2 *and subsection “*EEG manipulations*”.* Significance was defined as a P-value less than 0.05 for all statistical tests. In Figure 2, NS indicates no significant difference, ∗ indicates *p*-value < 0.05 and ∗∗ indicates *p*-value < 0.005.
