# Supplementary material for: EEG-based deep learning estimates preterm infants’ cerebral age: Contribution of slow-wave and cross-frequency coupling
Source: iScience. 2026 Oct 26;29(11):118052. doi: 10.1016/j.isci.2026.118052 (PMC13734788; doi:10.1016/j.isci.2026.118052)
Supplement: Supplementary file 1 [file mmc1.pdf]

iScience, Volume *29*

Supplemental information

EEG-based deep learning estimates preterm infants' cerebral age: Contribution of slow-wave and cross-frequency coupling

Alban Gallard, Alexander Billiot, Émilie Bourel-Ponchel, Pauline Brunel, Christèle Chazal, Guy Kongolo, Ghida Ghostine, Fabrice Wallois, Laura Routier, and Sahar Moghimi

Table S1: Clinical characteristics of the 165 newborns included in the study. Categorical features are expressed in number (percentage) and quantitative features in mean ± standard deviation.

Feature	Whole population	28-31 wGA subgroup	32-33 wGA subgroup	34-37 wGA subgroup
N	165	46	65	54
Gestational age at birth (weeks)	31.3 ± 1.9	29.4 ± 1.3	31.6 ± 1.1	32.4 ± 2.0
Age at EEG recording (wPMA)	33.1 ± 2.0	30.6 ± 1.1	33.1 ± 0.5	35.3 ± 1.0
Delay between birth and EEG recording (weeks)	1.9 ± 1.7	1.2 ± 0.7	1.5 ± 1.0	2.9 ± 2.4
Sex				
Female	87 (53%)	26 (57%)	28 (43%)	33 (61%)
Male	78 (47%)	20 (43%)	37 (57%)	21 (39%)
Delivery				
Vaginal delivery	73 (44%)	21 (46%)	31 (48%)	21 (39%)
C-section	92 (56%)	25 (54%)	34 (52%)	33 (61%)
Apgar				
1 min	8.07 ± 2.35	7.64 ± 2.81	8.23 ± 1.92	8.25 ± 2.34
5 min	8.99 ± 1.49	8.89 ± 1.88	9.09 ± 1.25	8.96 ± 1.41
Weight at birth (g)	1480 ± 390	1240 ± 240	1510 ± 350	1640 ± 440
Weight at recording (g)	1600 ± 390	1240 ± 250	1630 ± 260	1920 ± 350
Head Circumference at recording (cm)	28.5 ± 2.1	26.3 ± 1.7	28.7 ± 1.2	30.1 ± 1.6

n = number; wPMA = weeks postmenstrual age; wGA = weeks gestational age;

Table S2: Post-hoc Analysis. **UMAP**: Evaluation of the difference in age between each point and its 15 neighbours after each block. A one-way ANOVA on the mean age difference with the neighbours, with the factor block, was conducted and followed by a post-hoc analysis, providing evidence on progressive encoding over the blocks toward the prediction of cerebral age. **Prediction between groups**: To investigate prediction accuracy over each age group, a one-way ANOVA on the prediction errors with factor age was conducted. It was followed by a post-hoc analysis, providing evidence of the model's accuracy within each age range. **Scenario and age**: A 4 x 3 ANOVA with scenario as a within-subjects factor and age as between-subject factor was conducted to investigate the impact of modulations on the prediction of cerebral maturation. This was followed by a post-hoc analysis for the factor "scenario", carried out separately within each age subgroup proving evidence regarding the importance of each characteristic as a function of postmenstrual age.

Feature	M	CI	p
UMAP			
Block 1 and 2	2.82 ± 0.05	[2.65; 2.99]	< 0.0001
Block 1 and 3	-3.56 ± 0.05	[-3.74; -3.39]	< 0.0001
Block 1 and 4	-8.82 ± 0.05	[-8.99; -8.64]	< 0.0001
Block 2 and 3	-6.38 ± 0.05	[-6.56; -6.21]	< 0.0001
Block 2 and 4	-11.64 ± 0.05	[-11.81; -11.46]	< 0.0001
Block 3 and 4	-5.25 ± 0.05	[-5.43; -5.08]	< 0.0001
Prediction between groups			
Youngest - Middle	-0.50 ± 0.08	[-0.76; -0.24]	< 0.0001
Middle - Oldest	0.30 ± 0.07	[0.05; 0.55]	0.015
Youngest - Oldest	-0.20 ± 0.08	[-0.47; 0.07]	0.19
Scenario and age			
Youngest, Scenario 1	0.14 ± 0.16	[-0.32; 0.60]	0.54
Youngest, Scenario 2	1.11 ± 0.25	[0.39; 1.84]	0.0035
Youngest, Scenario 3	0.57 ± 0.18	[0.055; 1.09]	0.031
Middle, Scenario 1	0.10 ± 0.11	[-0.21; 0.41]	0.52
Middle, Scenario 2	1.04 ± 0.21	[0.44; 1.64]	0.00087
Middle, Scenario 3	0.45 ± 0.14	[0.05; 0.86]	0.028
Oldest, Scenario 1	0.05 ± 0.14	[-0.37; 0.48]	0.81
Oldest, Scenario 2	0.58 ± 0.18	[0.069; 1.09]	0.027
Oldest, Scenario 3	0.36 ± 0.17	[-0.12; 0.84]	0.14
